# Diagnostic value of routine CT perfusion imaging for radiology residents

**DOI:** 10.1038/s41598-024-76531-6

**Published:** 2024-10-23

**Authors:** Philip M. Nicolas, Ziad Maksoud, Nabila Gala Nacul, Burak Han Akkurt, Manoj Mannil, Manfred Musigmann

**Affiliations:** University Clinic of Radiology, University Hospital Münster, University of Münster, Albert-Schweitzer Campus 1, 48149 Münster, Germany

**Keywords:** CT-perfusion (CTP), Stroke, Cerebral, Ischemia, Diagnostic accuracy, Cell death in the nervous system, Diagnostic markers

## Abstract

To evaluate whether incorporating CT perfusion imaging can significantly enhance diagnostic CT accuracy in stroke detection. Two 3rd-year residents (3rd of 5 years of residency) reviewed CT scans of 200 patients with suspected stroke, consisting of 104 patients with a proven stroke and a control group with 96 patients. They analyzed each patient in a blinded and randomized manner in two runs. In one session, they had only non-contrast CT and CT angiography available for diagnosis; in the other session at a later time point, an additional CT perfusion imaging was available. The performance achieved by the two readers was determined in terms of AUC (area under the curve), accuracy, sensitivity, specificity, positive and negative predictive value and Cohen’s Kappa. Reader 1 achieved an AUC of 87.64% with the basic stroke-protocol vs. an AUC of 97.4% with an additional CT-perfusion given. Based on the DeLong test, these values differ significantly (p-value: 0.00017). Reader 2 achieved an AUC of 91.23% in basic stroke-protocol vs. an AUC of 96.42% with an additional CT-perfusion. These values also differ significantly (p-value: 0.02612).. The performance gain achieved with CT-perfusion is most evident in the decrease in the number of false classified cases (Reader 1: 24 to 5; Reader 2: 18 or 14 to 7) and the significant increase in Cohen’s kappa. Our study shows that additional CT-perfusion imaging in stroke diagnosis significantly improves the diagnostic reliability of residents. Therefore, it should be further investigated whether perfusion imaging should be a general standard of initial stroke diagnosis no matter of the onset.

## Introduction

Stroke is characterized as an acute injury to the central nervous system that results in neurological deficits, caused by vascular occlusion and/or hemorrhage^1^. With an incidence of 220/100,000 residents in Europe^2^ and a lifetime risk up to 24.9%^3^, it ranks among the most significant health issues globally.

Multimodal CT-imaging plays a central role in the diagnosis of stroke, including non-contrast CT (NCCT), CT-angiography (CTA) and in certain cases CT-perfusion (CTP)^4^. NCCT allows a fast and highly sensitive differentiation between hemorrhagical and ischemic^5^. Early signs of an ischemic stroke in the NCCT like a hyperdense-artery-sign or loss of grey-white matter differentiation require a high level of experience to detect and correctly interpretate^6^. In addition early changes within the basal ganglia may be apparent within the first hour only in 60% of patients with appropriate occlusion^5^. Furthermore, NCCT is not capable of excluding several stroke-like such as psychological disorders, migraine and seizures. The method that should be used for direct visualization of vascular occlusions is CTA^7^. It also allows assessment of proximally located vascular pathologies and visualization of collateral circulation^8^. An additional CTP provides information about the ischemic core and penumbra. Penumbra is defined as salvageable hypoperfused cerebral tissue^9^ and can be visualized as a mismatch between the infarct core (represented by the reduced cerebral blood volume (CBV)) and the critically hypoperfused area (represented by the reduced cerebral blood flow (CBF) in CTP.

Next to IV Thrombolysis the mechanical thrombectomy represents a causal therapy option for acute stroke. A favorable effect over standard care was demonstrated across all age groups in patients who underwent thrombectomy within 6 h of onset and had the following characteristics: proven causative vessel occlusion of the internal carotid artery or M1 segment, prestroke mRS 0–1, NIHSS ≥ 6, and ASPECTS ≥ 6^10^. This effect is shown in better disability outcomes at 90 days (mRS). Therefore, due to the 2019 Guidelines for Management of acute ischemic stroke (AIS), all patients who meet the criteria mentioned above should receive thrombectomy^10^. Thrombectomy should also be considered in patients with anterior, vertebral, basilar, or posterior occlusions, mRS > 1, NIHSS <, and ASPECTS < 6, although there is insufficient evidence of benefit based on current research^10^. In a period of 6–24 h since onset, for example in a wake-up situation, thrombectomy is recommended too, if a relevant mismatch is evident on perfusion imaging^11^. This makes CTP an important and helpful parameter of treatment decision-making^10^.

An efficient workflow with minimal delays is essential in the treatment of stroke because therapeutic success decreases rapidly over time^12^. In consequence every additional procedure like a CTP scan must outperform its delay, which is in median about 5.3 min^13^. According to the current state of research, it is recommended that stroke CT-imaging should consist of NCCT and CTA. In addition, CTP is performed if the onset of clinical symptoms is more than 4.5 h ago^7^. While most diagnostic stroke trials center upon the treatment decision and/ or patient benefit, few have investigated the radiologists’ point of view in diagnosing an ischemic stroke in multimodal CT.

The purpose of this study was to determine if CTP implemented in the standard diagnostic algorithm of acute ischemic stroke may enhance the sensitivity of Radiology residents under consideration of time delay and hazardous ionizing radiation.

## Material and methods

### Patient population

This retrospective single-center study was approved by the local ethics committee (Ärztekammer Westfalen-Lippe (ÄKWL), Münster 2022-297-f-S) and is in accordance with the Declaration of Helsinki. Informed consent was waiwed by the local ethics committee (Ärztekammer Westfalen-Lippe (ÄKWL). We searched the databases of our radiology information system for patients who received CT-graphic imaging according to the local stroke CT protocol with suspected stroke within the period from 04.10.2020 to 31.07.2021. To be included in the study cohort, (1) a clinically obtained NIHSS and (2) imaging with NCCT, CTA, and CTP (including CBV, CBF, and TTD) of good diagnostic quality had to be available. Two cohorts were created from the included cases. A group with proven stroke (n = 104) and a control group without stroke diagnosis (n = 96). In the stroke group, the mean age was 72.9 years (standard deviation 14.4 years), the mean NIHSS score was 13.0 (standard deviation 6.6), and the proportion of female was 55.3%. In 68.9% of these cases the gray-white matter differentiation was abolished, 66.0% of these cases showed a dens artery sign, and in 43.7% of these cases an M1 occlusion was present. The control group had a mean age of 75.2 years (standard deviation 14.9 years), a mean NIHSS score of 6.1 (standard deviation 4.4), and 59.4% of the patients were female.

### CT scan protocol

Imaging was performed on a second-generation dual-source CT scanner (Somatom Force Siemens Healthineers). The default stroke-protocol of our institute was used.

This protocol consists of a NCCT at the beginning, followed by an CTA and a CTP (detailed scan parameters in supplementary information). The reconstruction and computer-assisted evaluation was performed with syngo.via, Siemens Healthineers^®^. A TTD-, a CBV-, a CBF- and a MTT-map were calculated from the data set.

Acquisition of the entire CT stroke protocol requires an average of approximately 3650 mGycm in our institute. Thereof, approx. 1550 mGycm accrue for perfusion imaging, which corresponds to 42.5% of the total dose. The CTDIvol of the perfusion sequence is 104 ± 2 mGy.

The specified dose length product (given in mGycm) can be converted into the effective dose (given in mSv) using a mean weighting factor (mean values from male and female bodies). An average weighting factor of 0.0021 mSv/mGycm is used for examinations of the head. This results in an effective dose of 7.665 mSv for our entire stroke protocol. The CT perfusion fraction of this is 3.255 mSv.

### Clinical data analysis

Superior verified CT findings and patient records of all 200 patients were reported in a structured manner (P.N.). Age, sex, current symptoms leading to the suspected diagnosis of stroke, NIHSS, and, if present, dens artery sign, loss of grey-white matter differentiation, insular ribbon sign, vanishing basal ganglia, intracerebral vascular occlusions, perfusion deficits, and the ASPECT-score were noted. The diagnosis was verified by experienced senior neuroradiologists and by interventional angiography.

### Diagnosis of stroke

The diagnosis of stroke was made according to the standards of the American Heart Association or American Stroke Association^10^. Accordingly, the NIHSS was obtained at initial patient contact. Subsequently, imaging was performed to exclude intracerebral hemorrhage and to detect stroke-specific indicators like dense artery sign or loss of grey-white matter differentiation. In contrast to current recommendations, patients received perfusion imaging as a standard part of the CT stroke protocol rather than at an onset > 4.5 h. The diagnosis of stroke was made upon detection of a correlate on imaging that matched the clinical findings.

### Image analysis

All CTs of the entire study population were assessed by two blinded and independent readers (G.N., Z.M.). Each patient’s imaging was analyzed a total of two times. In addition to the clinical requirement, NCCT, CTA, and CTP were available in one run. In the other run, only NCCT and CTA were available. The order, of the runs was random. There were 10 weeks between the two runs. The two readers analyzed the imaging for the presence of stroke. They noted early infarct signs in NCCT if present. In addition, they analyzed whether a vessel occlusion was present. If a CTP was provided in the run, they also noted whether a perfusion deficit was present.

### Statistical analysis

Statistical analysis was performed using R software (version 4.1.2). The performance achieved by the two readers was determined in terms of AUC (area under the curve), accuracy, sensitivity, specificity, positive and negative predictive value and Cohen’s Kappa. In the context analysed, sensitivity means the correct prediction of cases with a stroke, while specificity means the correct prediction of cases without a stroke. The positive predictive value describes the proportion of correctly predicted cases with a stroke in relation to all predicted cases with a stroke. Accordingly, the negative predictive value describes the rate of correct predictions of cases without a stroke in relation to all predictions of cases without a stroke. Finally, Cohen’s Kappa is defined as: (observed accuracy − expected accuracy)/(1 − expected accuracy)). The Friedman test and subsequent pairwise Wilcoxon signed-rank tests were used to test whether the groups (true outcome and readers) differed significantly. In addition, we used the DeLong test^14^ to compare the AUC values obtained with a basic stroke CT-protocol, including NCCT and CTA and a protocol using an additional CT-perfusion.

## Results

**Fig. 1:**
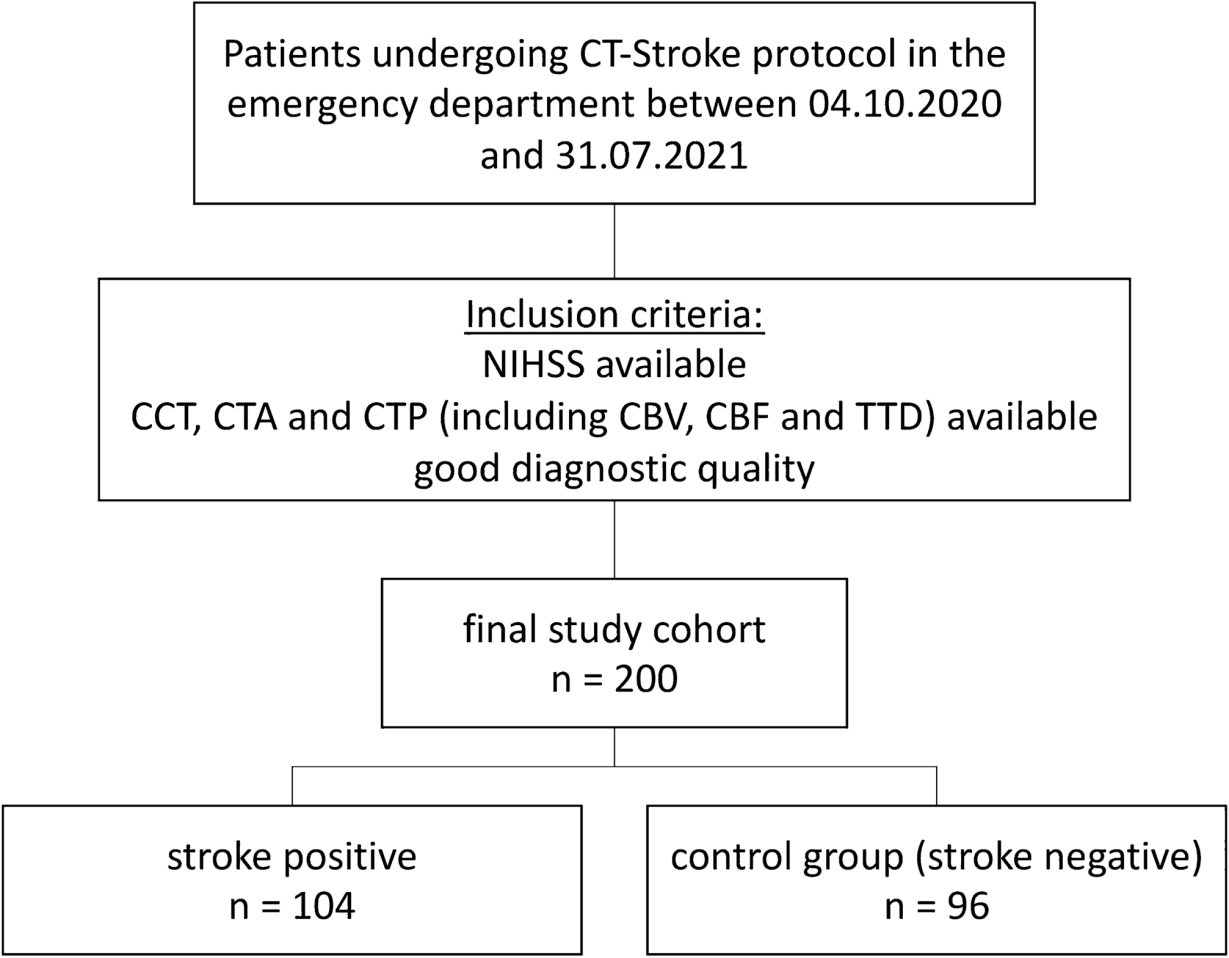
Patient population.

First, we calculated the means of the true outcome and the two independent readers in predicting true strokes. The results are summarized in Table 1. Following Fig. 1, we included 104 patients with a true stroke and 96 patients without a stroke in our study, resulting in a proportion of cases with true strokes of 52.0%. Since peripheral vascular occlusions are more difficult to diagnose, we also performed a subgroup analysis in which the central (M1) occlusions were excluded. In this subgroup, the proportion of cases with true strokes is 38.1%.

**Table 1:** Proportions of true (outcome) and predicted (Reader 1 und 2) strokes.

	All cases included		M1 strokes excluded	
	Basic stroke CT (%)	Additional CTP (%)	Basic stroke CT (%)	Additional CTP (%)
True outcome	52.0	52.0	38.1	38.1
Reader 1	60.0	54.5	48.4	41.3
Reader 2	44.0	53.5	30.3	40.0

As shown in Table 1, Reader 1 tends to over-diagnose strokes, while Reader 2 under-diagnoses them. For instance, Reader 1 diagnoses a stroke in 60% of cases based on a basic stroke CT, even though a stroke is present in only 52% of cases. On the other hand, Reader 2 identifies a stroke in just 44% of the same cases. Interestingly, in both scenarios, whether M1 strokes are included or not, the results from CT perfusion imaging are much closer to the actual outcomes. To assess whether there was a significant effect, we conducted a Friedman test followed by pairwise Wilcoxon signed-rank tests for pairwise comparisons. We began by comparing the three groups (true outcome, Reader 1, and Reader 2) using a basic stroke CT protocol, which included NCCT and CTA. In both the full dataset and after excluding M1 cases, the three groups, as well as all pairwise combinations, showed highly significant differences. The largest p-value, 0.002, was observed when comparing Reader 2 with the true outcome after excluding M1 cases. All other comparisons resulted in p-values of ≤ 0.001, indicating substantial differences between the groups. We then repeated the analysis using an extended stroke protocol that included CT perfusion. In this case, no pairwise comparisons, whether M1 cases were excluded or not, showed a significant difference. The deviations between the results are significantly smaller in the case of CT-perfusion than in the case of basic stroke CT- imaging.

**Fig. 2:**
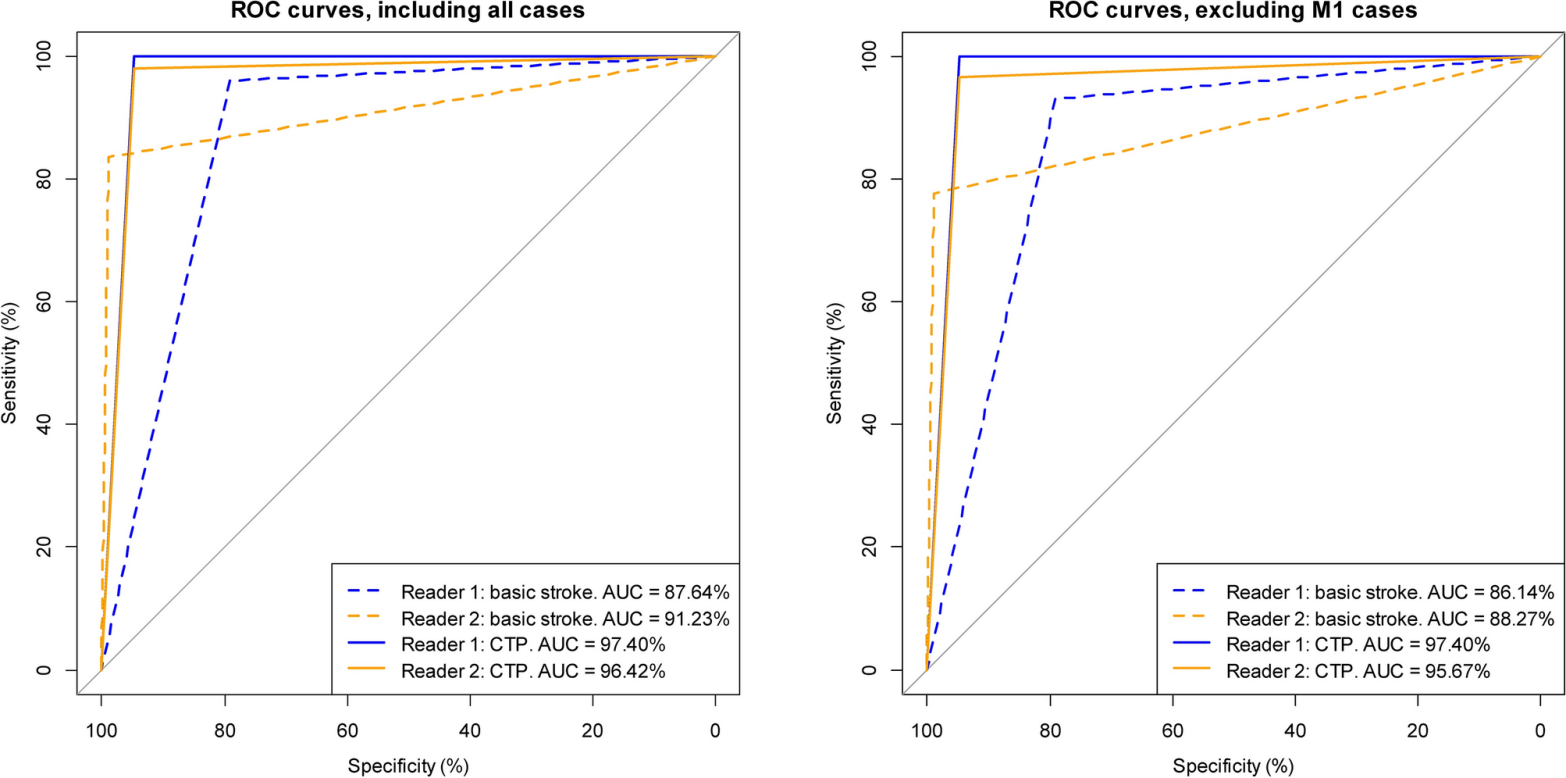
ROC curves including all cases (left figure) and excluding the M1 cases (right figure). The dashed lines were calculated from basic stroke CT-imaging and the solid lines from CT-perfusion (CTP).

The calculated AUC values confirm this first impression that the discriminatory power can be significantly increased by using CT-perfusion. The corresponding ROC curves (Reader 1 = blue lines, Reader 2 = orange lines), calculated with all cases as well as excluding the M1 cases are shown in Fig. 2. The dashed lines refer to basic stroke CT-imaging and the solid lines to CT-perfusion. The AUC values shown in the figures indicate a considerable increase in the discriminatory power achieved. To test whether the ROC curves/AUC values (basic stroke CT-protocol and additional CT perfusion) are indeed significantly different, we performed the DeLong test. Including all cases resulted in a p-value of 0.00017 for Reader 1 and a p-value of 0.02612 for Reader 2. Excluding the M1 cases, we obtained p-values of 0.00014 and 0.02428, respectively. This means that the AUC values for both readers have demonstrably increased significantly (both including and excluding M1 cases). Especially for Reader 1, the use of CT-perfusion results in a significant increase in discriminatory power. However, the increase in discriminatory power is also significant in case of Reader 2.

In addition to the AUC values shown in Fig. 2, we calculated the accuracy, sensitivity, specificity, positive and negative predictive value, Cohen’s Kappa and the total number of false classified cases. The results including all cases are summarized in Table 2. Table 3 shows the corresponding results for the case in which M1 strokes were excluded. With the exception of the specificity and positive predictive value for Reader 2, all performance measures show an increase in discriminatory power when using CT-perfusion. The performance gain achieved with CT-perfusion is most evident in the decrease in the number of misclassified cases (i.e. false-positive and false-negative classified cases) and the significant increase in Cohen’s kappa. In the case of Reader 1, the number of false classified cases dropped from 24 to only 5, a decrease of 79.2%. In case of Reader 2, on the other hand, the number of misclassified cases decreased from 18 and 14 to 7. Here, the error rate was reduced by 61.1% and 50.0% respectively. Most importantly, CT-perfusion has a higher sensitivity. Strokes that were not detected with basic stroke CT-imaging could be correctly diagnosed with CT-perfusion. Tables 2 and 3 also include the 95% confidence intervals (CI) for the accuracy values. The accuracy values obtained with basic stroke CT-scans are outside or at the absolute margins of the 95% confidence intervals of the accuracy values obtained when additional CT-perfusion imaging is performed. For example, according to Table 2, Reader 1 has an accuracy of 87.94% if only basic stroke CT-scans are performed, but his accuracy is between 94.23% and 99.18% with a 95% probability (95% CI) if CT-perfusion imaging is also performed. Overall, the results obtained with CT-perfusion show significantly higher discriminatory power than those obtained with basic stroke CT-imaging.

**Table 2:** Performance values received with basic stroke CT-imaging and CT-perfusion, including all cases. The AUC values obtained with and without CT-perfusion imaging differ significantly (p values: 0.00017 and 0.02612 respectively, based on the DeLong test) for both Reader 1 and Reader 2. CI = confidence interval.

Performance measure	Reader 1		Reader 2	
	Basic stroke CT	Additional CTP	Basic stroke CT	Additional CTP
AUC	87.64%	97.40%	91.23%	96.42%
Accuracy	87.94%	97.49%	90.95%	96.48%
Accuracy 95% CI	(82.59%, 92.12%)	(94.23%, 99.18%)	(86.08%, 94.55%)	(92.89%, 98.57%)
Sensitivity	96.12%	100.0%	83.50%	98.06%
Specificity	79.17%	94.79%	98.96%	94.79%
Pos. predictive value	83.19%	95.37%	98.85%	95.28%
Neg. predictive value	95.00%	100.0%	84.82%	97.85%
Cohen’s Kappa	75.71%	94.96%	81.99%	92.95%
Total number of misclass. cases	24	5	18	7

**Table 3:** Performance values received with basic stroke CT-imaging and CT-perfusion, M1 cases excluded. The AUC values obtained with and without CT-perfusion imaging differ significantly (p values: 0.00014 and 0.02428 respectively, based on the DeLong test) for both Reader 1 and Reader 2. CI = confidence interval.

Performance measure	Reader 1		Reader 2	
	Basic stroke CT	Additional CTP	Basic stroke CT	Additional CTP
AUC	86.14%	97.40%	88.27%	95.67%
Accuracy	84.42%	96.75%	90.91%	95.45%
Accuracy 95% CI	(77.70%, 89.75%)	(92.59%, 98.94%)	(85.22%, 94.94%)	(90.86%, 98.15%)
Sensitivity	93.10%	100.0%	77.59%	96.55%
Specificity	79.17%	94.79%	98.96%	94.79%
Pos. predictive value	72.97%	92.06%	97.83%	91.80%
Neg. predictive value	95.00%	100.0%	87.96%	97.85%
Cohen’s Kappa	68.53%	93.20%	79.81%	90.42%
Total number of misclass. cases	24	5	14	7

**Fig. 3:**
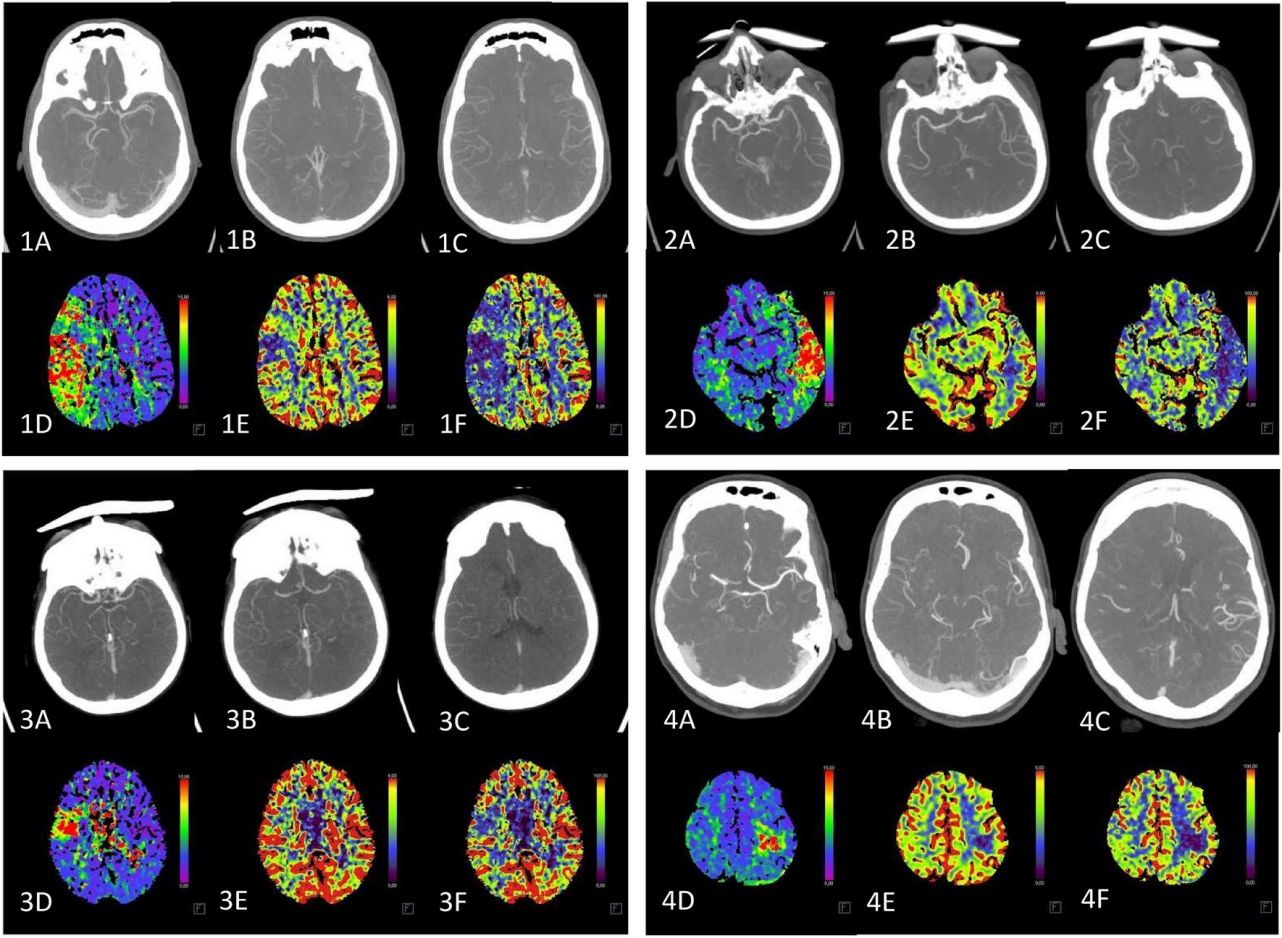
The higher sensitivity of perfusion-Imaging (D-F) compared to CTA (**A**–**C**). The CTA is shown representatively as MIP (maximum intensity projection) of the relevant vascular regions. In the CTA, no vessel occlusion is visible although there appears a territorial perfusion deficiency in the TTD (**D**) as well as a mismatch in CBV (**E**) and CBF (**F**).

The benefits of perfusion imaging can be illustrated in Fig. 3. A clear territorial perfusion deficit is evident, but no vessel occlusion can be delineated. This fact was verified by a senior physician and is represented by a MIP (maximum intensity projection) of the relevant vascular regions.

## Discussion

It has already been demonstrated that multimodality CT diagnostics including CT perfusion has a significantly higher stroke detection rate compared with NCCT, CTA, or CT perfusion alone^15^.

With our study, we are able to demonstrate that CT perfusion provides a significant improvement in the diagnostic power of younger residents, improving both sensitivity and specificity. Especially for more difficult to diagnose vascular occlusions such as peripheral occlusion or in the posterior circulation, an improvement in diagnostic confidence could be shown.

Current guidelines recommend the use of perfusion imaging only after 6 h from stroke onset (such as in typical wake-up strokes) to better assess the appropriateness of mechanical thrombectomy. Most analyses are based on cases evaluated by experienced specialists with many years of practice. However, this setup does not reflect real-world conditions, where stroke diagnostics in emergency settings are often performed by less experienced residents, particularly during night shifts. In this context, we demonstrated a significant benefit of adding perfusion imaging, which substantially increased diagnostic confidence and significantly reduced the rate of misdiagnosis.

Another widely discussed topic is the value of CT versus MRI imaging in acute stroke diagnostics. Especially within the first 24 h, the sensitivity of CT imaging, which is still the standard due to its good availability, short acquisition time, and cost–benefit ratio, is clearly inferior to MRI. However, this disadvantage can be adjusted by additional perfusion imaging^16^.

A key difference between our study and previous ones is that we selected a patient cohort that closely reflects real-world conditions, without restricting the study to anterior circulation occlusions. This is important because perfusion imaging is particularly valuable in diagnosing more challenging occlusions, such as peripheral vessel occlusions and posterior circulation occlusions. In our cohort, 10 patients had posterior circulation occlusions, and 31 had peripheral occlusions (M2-4, A2) of the anterior circulation. This benefit was particularly evident in the subanalysis (excluding M1 occlusions) for Reader 1. To further investigate the advantage of CTP in diagnosing peripheral and posterior circulation occlusions, a follow-up study with a higher proportion of these cases should be considered. Our results are consistent with the findings of Alotaibi et al., who were also able to show that additional perfusion imaging leads to a significant improvement in diagnostic power. Occlusions of the posterior circulation or peripheral occlusions in particular often represent a challenge for young residents. Here, the additional benefit of CTP was particularly high, as our sub-analysis was able to show. Alotaibi et al. were also able to show that there is a significant reduction in the time required for diagnosis with the use of CTP. This results in more efficient patient care and therefore a better outcome (10.1177/23969873231214218).

A critical aspect regarding an additional perfusion imaging is the added radiation exposure that has to be applied for this purpose. However, in our case, our team of medical physicists succeeded in reducing the additional radiation exposure (CTDIvol) to only 104 ± 2 mGy, resulting in an additional radiation exposure of 42.5%. As mentioned above this equals an additional effective dose of 3.255 mSv.

In order to be able to better estimate the effective dose, it is worth comparing it with the natural annual radiation exposure. In Germany, this is an average of 2.1 mSv per year, whereby these values can vary from 1.0 to 10 mSv depending on lifestyle and place of residence (source: Federal Office for Radiation Protection in Germany, https://www.bfs.de/DE/themen/ion/umwelt/natuerliche-strahlung/natuerliche-strahlung_node.html). A CT perfusion therefore corresponds to an average radiation exposure of approx. 1.55 years.

The main danger of ionising radiation is stochastic radiation damage with consecutive damage to DNA, which can lead to mutations and malignancies. The lifetime cancer mortality risk can be used to estimate the additional risk. The additional individual, relative lifetime cancer mortality risk due to ionising radiation from whole-body exposure with a single dose is estimated at a total of 5% per sievert (10.3238/arztebl.m2022.0395). Converted to the average radiation exposure of a CT perfusion, this results in an additional lifetime cancer mortality risk of 0.0163%.

Another example of assessing the risk of ionizing radiation is the reference limit for the protection of occupationally exposed individuals. These individuals may receive up to 20 mSv per year or 400 mSv over their working lifetime, which is significantly higher than the dose used in a typical stroke CT scan.

Considering the significant reduction in misdiagnosis, we consider this additional radiation exposure to be justified in our typical patient cohort.

For younger patients, who face a higher risk of radiation-related harm due to their longer remaining lifespan, it is crucial to minimize radiation exposure. While we successfully reduced the additional radiation from CTP, the potential benefits must be carefully weighed against the risk of radiation damage in this group. Alternatives include using MRI or initially performing only an NCCT and CTA, reserving CTP for cases where there is diagnostic uncertainty.

Our study has certain limitations, as CT perfusion was initially tested on only two residents at the same stage of their training. For more comprehensive analysis, it would be beneficial to include a larger group of readers with varying levels of experience. A comparison with senior physician benchmarks would also add value. Additionally, the retrospective nature of the study introduces inherent limitations.

In our study, we included only examinations with good diagnostic quality. However, in real-world practice, achieving optimal diagnostic quality is not always feasible due to factors like motion artifacts or prolonged cycle times. In cases where a CTA is compromised by motion, a CTP could help confirm the suspicion of a vascular occlusion. Conversely, if the CTP is non-diagnostic due to excessively slow cycle time, it would result in unnecessary additional radiation exposure without any diagnostic benefit.

## Conclusion

Our study demonstrated that CT perfusion can significantly improve sensitivity and specificity, particularly for younger physicians who have not yet attained specialist status but are often responsible for emergency diagnostics, such as during night shifts. In challenging cases, such as peripheral vascular occlusions or posterior circulation ischemia, diagnostic quality was notably enhanced. This raises the question of whether perfusion imaging should become a standard component of initial stroke diagnosis, regardless of the time since onset, and warrants further investigation.

## Supplementary Information

Supplementary Information.

## Data Availability

The dataset generated and analyzed during the current study is available via Google Drive (filename: Stroke Study). Weblink: https://docs.google.com/spreadsheets/d/1LJVCZP6wNGOE3KW2SR5I3vRNi2uEwErA/edit?usp=sharing&ouid=106658788932018418702&rtpof=true&sd=true.
