# Supplementary material for: Diagnostic value of routine CT perfusion imaging for radiology residents
Source: Sci Rep. 2024 Oct 23;14:25093. doi: 10.1038/s41598-024-76531-6 (PMC11499635; doi:10.1038/s41598-024-76531-6)
Supplement: Supplementary file 1 — Supplementary Information. [file 41598_2024_76531_MOESM1_ESM.docx]

NCCT: effective mAs 332, kV 120, slice thickness 5.0 mm, slice acquisition 192 x 0.6 mm, scan direction caudocranial and a pitch of 0.55. The images were reconstructed in three ways: at a Hr40 kernel (with a strength level of 2, a slice thickness of 5.0 mm and a slice increment of 5.0 mm), a Hr32 kernel (with a strength level of 1, a slice thickness of 1.0 mm and a slice increment of 0.6 mm) and a Hr59 kernel (with a strength level of 1, a slice thickness of 1.0 mm and a slice increment of 0.6 mm).

Contrast material injection for CTA: A bolus of 60 ml nonionic iodinated contrast material (Ultravist 370) is injected, at a flow rate of 4.0 ml/s, followed by a European Radiology saline flush of 50 ml at a flow rate of 4.0 ml/s. The scan initiation was triggered by a bolus tracking using a ROI in the pulmonary trunk, aiming for 120 Hounsfield units (HU).

CTA: kV 100, slice thickness 1.0 mm, slice acquisition 128 x 0.6 mm, scan direction caudocranial and a pitch of 1.15. An automatic tube current modulation (CareDose4D, Siemens Healthineers) was used. The images were reconstructed in two ways: at a Hv36 kernel (with a strength of 2, a slice thickness of 1.0 mm and an increment of 0.6 mm) and a Hr59 kernel (with a strength of 1, a slice thickness of 1.0 mm and an increment of 0.6 mm).

Contrast material injection for CTP: A bolus of 30 ml nonionic iodinated contrast material (Imeron 350) is injected at a flow rate of 5.0 ml/s, followed by a European Radiology saline flush of 50 ml at a flow rate of 5.0 ml/s.

CTP (DynMulti4D): Scan start is injector coupled via a manual button. mAs 170, kV 70, slice thickness 10.0 mm, slice acquisition 48 x 1.2 mm, 4D Range 114 mm 1.5 s, scan direction caudocranial. The images were reconstructed in two ways: at a Hr36 kernel (with a slice thickness of 10.0 mm and an increment of 4.0 mm) and a Hr36 kernel (with a slice thickness of 1.5 mm and an increment of 1.0 mm). The reconstruction and computer-assisted evaluation was performed with syngo.via, Siemens Healthineers ®.
